# Rothia dentocariosa endocarditis with brain abscess and splenic abscess: case report and brief review

**DOI:** 10.3389/fcvm.2024.1370736

**Published:** 2024-06-20

**Authors:** Xue Zheng, Fang Liu, Qiaoli Ma, Jing Li, Huiping Ma

**Affiliations:** ^1^ Clinical Pharmacy, Binzhou Medical University Affiliated Zibo Central Hospital, Zibo, Shandong, China; ^2^ Clinical Pharmacy, Shengli Oilfield Central Hospital, Dongying, Shandong, China; ^3^ Department of Cardiology, Binzhou Medical University Affiliated Zibo Central Hospital, Zibo, Shandong, China

**Keywords:** Rothia dentocariosa, endocarditis, brain abscess, spleen abscess, aortic valve replacement

## Abstract

Rothia dentocariosa is a conditionally pathogenic bacterium that may cause infective endocarditis (IE) in selected patients and give rise to a variety of clinical complications, albeit it is not a common IE pathogen. We present the case of a patient diagnosed with Rothia dentocariosa-associated IE secondary to influenza B and thrombocytopenic purpura. The blood culture revealed Rochebacterium caries, cardiac ultrasound detected vegetation, while brain and spleen abscesses manifested and progressively deteriorated. Despite a suboptimal response to anti-infective therapy, the patient ultimately underwent aortic valve replacement. Discharge from the hospital was achieved upon control of the brain abscess and spleen abscess.

## Introduction

Rothia dentocariosa is a common gram-positive bacterium first isolated from dental caries in 1967 (1), primarily colonizing the human oropharynx and respiratory tract as well as dental surfaces and gingival plaque (2, 3). It is considered an opportunistic pathogen with low virulence, leading to relatively rare infections that are rarely reported. Infective endocarditis (IE) is the most frequently associated infection. Other infections caused by R. dentocariosa include bacteremia, sepsis, meningitis, bone and joint infections, endophthalmitis, skin and soft tissue infections, peritoneal dialysis-related peritonitis, etc (4–6). To date, only over 30 cases of IE caused by R. dentocariosa have been reported worldwidely (6–36). The latest systematic literature analysis about Rothia spp IE by Franconieri F (36) had shown aortic valves(60%) and mitral valves(46%) are the most often affected valves in instances of Rothia dentocariosa endocarditis (36). A total of 64% of patients would experience complications (36). These included neurological complications [including but not limited to cerebral embolism (6, 25, 28–30), cerebral hemorrhage (6, 11, 29), cerebellar hemorrhage (8, 24, 25, 29), intracranial hemorrhages (24), intracranial mycotic aneurysms (8, 23), brain abscesses (7, 13, 24)], cardiac/endovascular (including but not limited to aortic root abscess (9, 15, 17, 23), papillary muscle dysfunction (34), artery abdominal mycotic aneurysms (12, 15), Mycotic aneurysm on right deep femoral artery (21), visceral infarcts/ abscesses(splenic infarction (25), renal infarction (25), and others (spinal osteomyelitis (6, 19), endophthalmitis (28). Despite these numerous complications, the mortality rate is still quite low, ranging from 12% to 14% (20, 29, 36).

## Case presentation

Our patient was a 40-year-old male without a particular previous medical history. On January 30, 2022, the patient was infected with the influenza B virus and then suffered from secondary lung infection, respiratory failure, and thrombocytopenic purpura. He underwent treatment of paramivir + oseltamivir, piperacillin-tazobactam and gamma globulin combined with high-dose glucocorticoid (80 mg/d of methylprednisolone for three days). After his condition improved, the patient was transferred from the superior hospital to our hospital on February 20, 2022.

**Figure 1:**
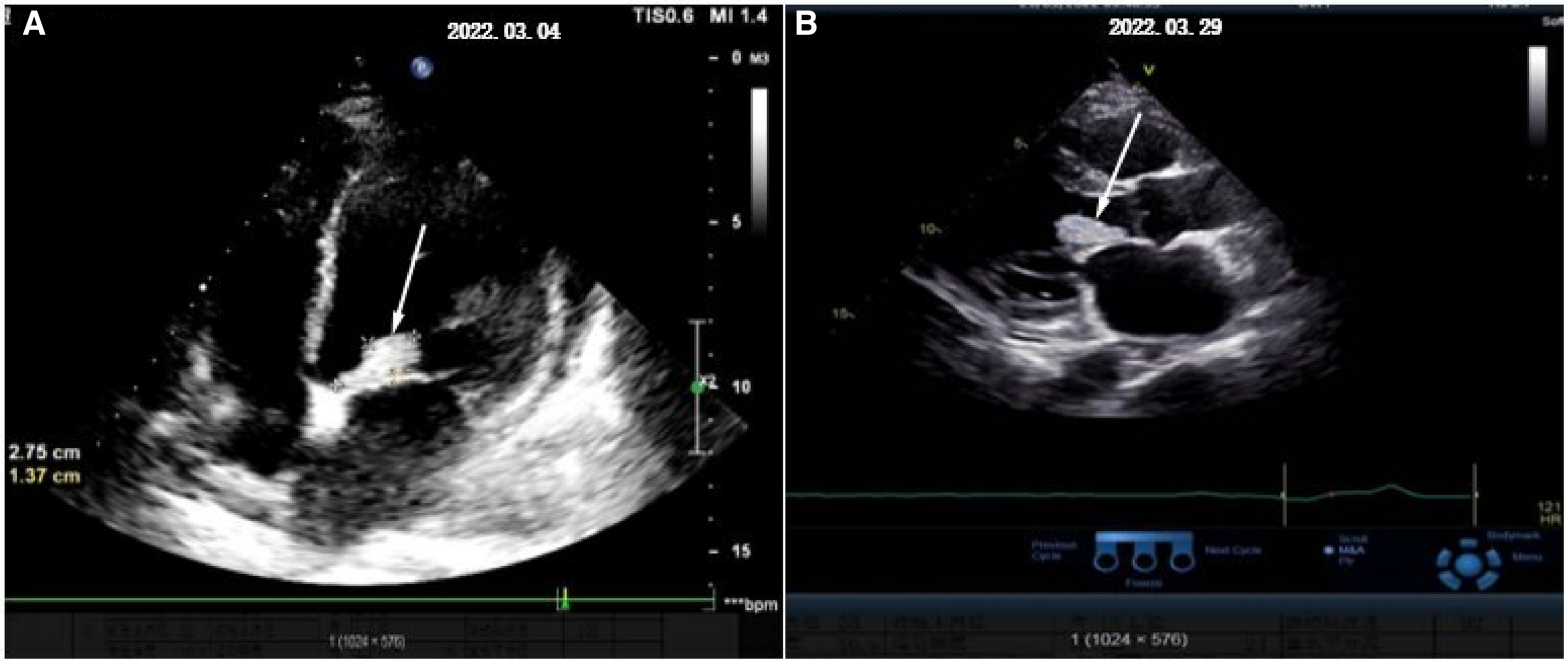
The development process of cardiac vegetations (denoted by white arrow). (**A)** Shows the initial discovery of the aortic valve vegetation and (**B)** shows what it looks like before surgery.

Vital signs on admission were a blood pressure of 135/80 mmHg, a pulse rate of 110 beats/min, a respiratory rate of 22/min, and an axillary temperature of 38.5°C. Laboratory data were significant for a white blood cells (WBC) count of 12.65*10^9^/L, neutrophils (percentage) of 94.2%, a C-reactive protein (CRP) concentration of 152.63 mg/L, a platelet count of 146*10^9^/L, and an erythrocyte sedimentation rate of 87 mm/h. The patient experienced tingling when he opened his eyes, but he was unable to communicate verbally. His right limb displayed decorticated flexion while his left limb did not exhibit any tingling response. The GCS score was six and a Comma was diagnosed. In addition, the unfortunate patient had several lingual and oral ulcers. He received minocycline 100 mg q12h for his bacterial lung infection. However, he had a persistent fever with a maximum body temperature of 39°C. On February 26 (day 7 of admission), blood culture results showed Rothia dentocariosa. Cardiac ultrasound (Figure 1A) identified a 28 × 14 mm isoechoic mass attached to the left coronary leaflet of the aortic valve swinging with the cardiac cycle (entering the aorta in systole and the left ventricle in diastole) and moderate aortic valve insufficiency. The patient was started on penicillin 3.2 million U every 6 h and amikacin 0.75 g every 12 h.

**Figure 2:**
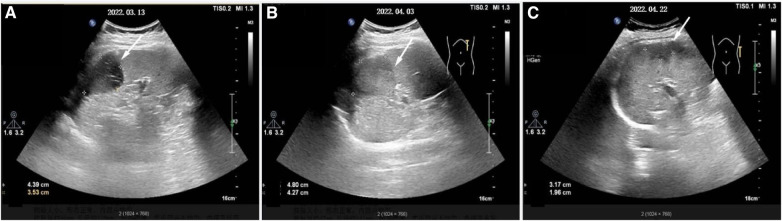
The development process of splenic abscess(denoted by white arrow). (**A–C**) Represent respectively the morphology of the splenic abscess when it is found at first, before surgery, and before discharge.

**Figure 3:**
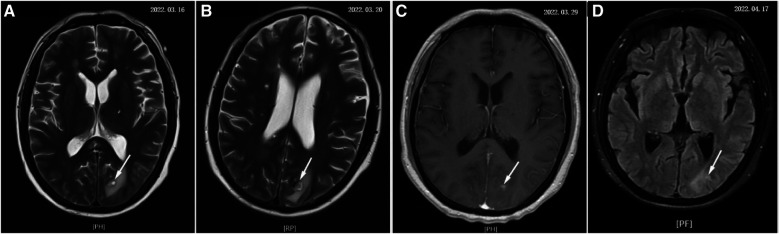
The development process of brain abscess (denoted by white arrow). (**A–D**) Show the changes of brain abscess throughout the course of the disease separately.

After that, the patient still had intermittent fever, and there wasn't a significant decrease in WBC and CRP. Abdominal ultrasound (Figure 2A) showed multiple cystic solid masses in the spleen measuring approximately 39 × 23 mm and 31 × 19 mm, which were considered to be splenic abscesses. Brain MRI (Figure 3A/B) showed an abnormal enhancement lesion in the left occipital lobe with a large oedema zone around it, and a small abscess was initially considered. The penicillin was discontinued, and ceftriaxone 2.0 g every 12 h and rifampicin 0.3 g every 6 h were started. The patient still had an intermittent fever. Vancomycin 1.0 g every 12 h and meropenem 1.0 g every 8 h were started, and ceftriaxone combined with rifampicin was discontinued on March 20 (day 29 of admission). The patient's body temperature dropped to 36.8°C, and had no fever thereafter. The WBC and CRP gradually decreased to the normal range. During the treatment process, fasting blood glucose was slightly elevated (6–8 mmol/L), liver and kidney function remained normal, and albumin was maintained at 30–40 g/L.

**Figure 4:**
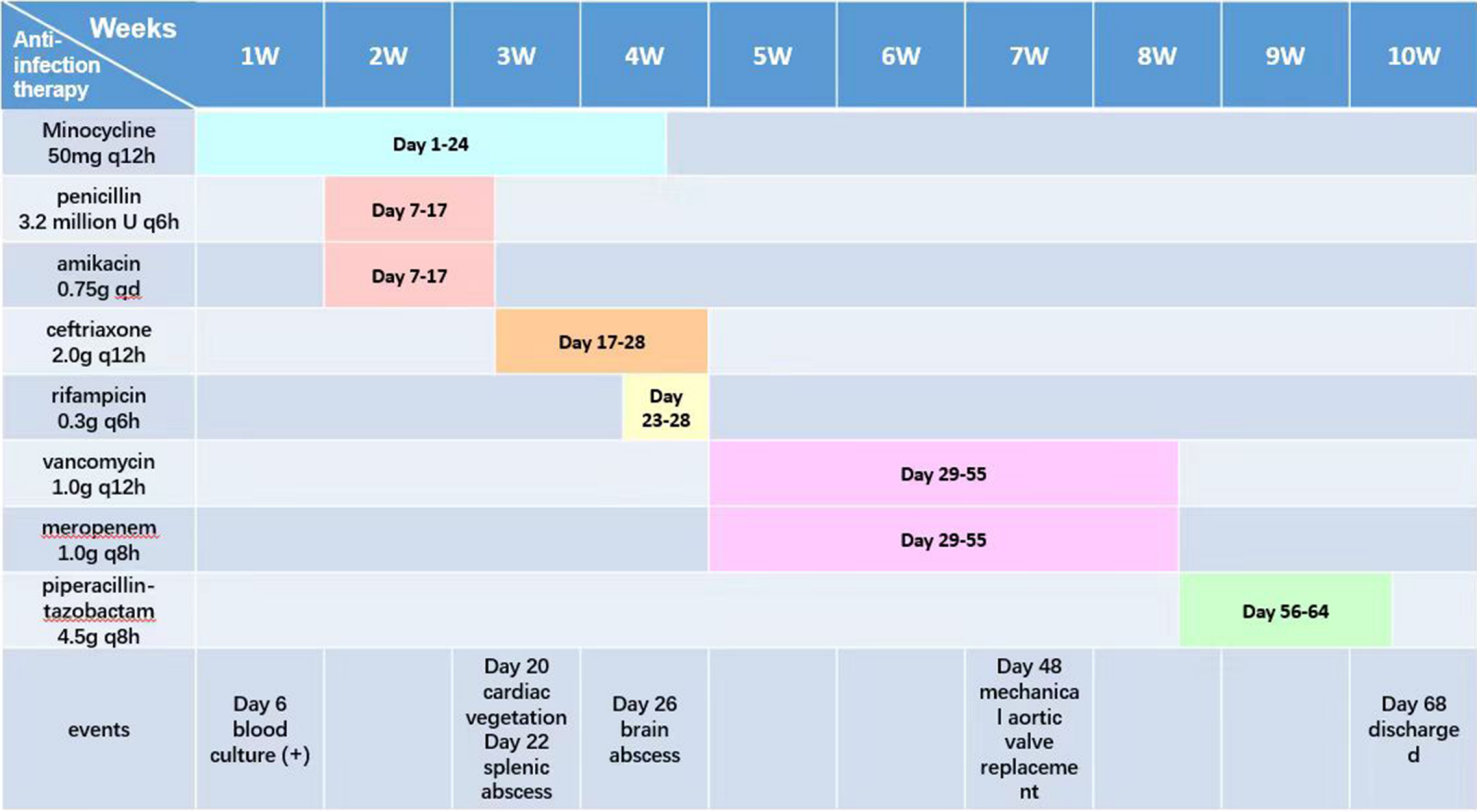
Detailed treatment schedule.

On March 29 (day 38 of admission), cardiac ultrasound showed that the echo mass of the aortic valve was approximately 25 × 14 mm (Figure 1B), slightly smaller the initially evaluated. Abdominal ultrasound showed several hypoechoic masses in the spleen, the largest of which was approximately 48 × 43 mm (Figure 2B). A review of the cranial MRI (Figure 3C) showed an abnormal enhancement lesion (2.72 × 3.92 mm) in the left occipital lobe, which was smaller than the anterior one. A new similar abnormal signal was observed nearby, indicating a high probability of an abscess. According to the latest results, the size of the aortic valve vegetation did not change, and the brain abscess and spleen abscess continued to progress, so the patient was transferred to cardiac surgery, and a mechanical aortic valve replacement was carried out under general anesthesia on April 8 (day 48 of admission). Following the procedure, the patient was still taking 1.0 g of vancomycin every 12 h along with 1.0 g of meropenem every 8 h. And nine days later (day 57 of admission), they were replaced by piperacillin-tazobactam, 4.5 g every 8 h. The abnormal enhancement lesion in the left occipital lobe (Figure 3D) was smaller (1.91 × 0.5 mm) than the anterior imaging. On April 20, the abdominal ultrasound revealed multiple low-density lesions in the spleen, with the largest being 32 × 20 mm (Figure 2C), which is considerably less than the original assessment. April 28th, day 68 of admission, marked the patient's final discharge. A figure (Figure 4) was added to display the timetable and relevant information from the patients' thorough therapy.

## Discussion

The genus “Rothia” was first described in 1967 by Georg and Brown (1). In 1978 the first incidence of endocarditis caused by Rothia dentocariosa was documented (7). Thirty-four instances were found when searching medline for R. dentocariosa IE (6–36). The most recent systematic literature review on Rothia spp IE, conducted by Franconieri F (36), included information on the disease's epidemiological characteristics, clinical characteristics, course of treatment, and results. We revised Table 1's data.

**Table 1:** Clinical characteristics of patients with Rothia dentocariosa endocarditis (*n* = 35).

Clinical feature	Proportion of patients	Percentage of patients
Demographic data		
Male	25	71.4%
Median age [range] (years)	46 [6–71]	
Comorbidities		
Pre-existing valvular disease	22	62.9%
Buccodental abnormalities	16	45.7%
IVDU	4	11.4%
Immunodeficiency	8	22.9%
Involved valves		
Native valve	28	80.0%
Prosthetic valve	7	20.0%
Aortic valve	19	54.3%
Mitralvalve	14	40.0%
Tricuspid valve	1	2.8%
Other	4	11.2%
Complications		
Neurological	22	62.9%
Cardiac/endovascular	8	22.9%
Extracerebral embolism	4	11.4%
Visceral abscess/abcess (spleen, kidney)	3	8.57%
Other^a^	3	8.57%
Treatmen		
Median duration of antibiotherapy [range] (weeks)	6 [2–13]	
Surgery	16	45.7%
Death at 6 months	4	11.4%

^a^ Vertebral osteomyelitis, endogenous endophthalmitis.

The primary risk factor for Rothia dentocariosa IE is structural valve disease, much as IE brought on by other gram-positive bacteria. The aforementioned table indicates that 62.9% (22/35) of patients have structural valve disease and 45.7% (16/35) have oral system disease. The individuals on the list also have other risk factors, such as diabetes, hypertension, hepatobiliary disorders, high hormone dosages, and elevated neutrophils. Nevertheless, it should be highlighted that infections with Rothia dentocariosa can develop even in the absence of known risk factors. Our patient was healthy without diabetes, alcoholism, liver disease, or valvular heart disease, and he also denied oral diseases, oral procedures, or dental surgeries. Unfortunately, the patient was suffering from influenza B virus infection, thrombocytopenic purpura, high-dose hormone therapy (80 mg/d of methylprednisolone for three days), and low immune function in the body. Even worse, he developed large ulcers on the lingual and oral surfaces, indicating that Rothia dentocariosa may have entered the circulation through the ulcer surface and ultimately resulted in IE.

80.0% of the patients had a native heart valve impacted, most often the mitral (40.0%) and aortic (54.3%) valves. Two of the valves were impacted in three individuals. Only one patient had three valves involved. Systemic complications occurred in 77.1% of cases, higher than reported (36). The most frequent problems are still neurological (62.9%) and cardiovascular (22.9%). Notably, seemingly minor side effects such spondylitis, endophthalmitis, and visceral infarction/abscess actually turn out to be more common. Notwithstanding the absence of a comparator group from the same period and location, the six-month mortality rate of Rothia dentocariosa IE appears to be lower than that of traditional Gram-positive cocci endocarditis (11.4% vs. 30%–37%) (37, 38).

Up to 35% of IE patients experience symptomatic cerebrovascular consequences (39, 40), whereas up to 80% of patients experience silent cerebrovascular complications, such as ischaemia and microhaemorrhage (41, 42). Although the clinical presentation might vary, the most frequent presentations are transient ischemic attacks and ischemic strokes (43). Other manifestations include hemorrhage (intracerebral, subarachnoid), meningitis, brain abscess, encephalopathy, and infectious aneurysms. Brain abscesses (BA) are severe lesions in the course of IE, accounting for approximately 5%–7% (44). Our patient is presently the fourth person to suffer from a Rothia dentocariosa-related brain abscess. With this case, the overall number of R. dentocariosa IE complicated by brain abscess is increased to 12.9% of the cases that have been recorded.

Preventing brain abscesses depends critically on early antibiotic medication commencement and prompt detection of IE. However, the course of treatment is lengthier and ought to go at least six to eight weeks (44). For BA larger than 10 mm in diameter, a standard antibiotic course of 10 weeks is recommended. For bigger BA (≥2.5 cm), those who do not improve with antimicrobial therapy after 1–2 weeks, and in cases of hydrocephalus or notable mass effect, neurosurgery is advised (44). For this patient, the treatment for infection showed impressive results, with the brain abscess rapidly shrinking. The brain abscess was decreased to 2.72 × 3.92 mm before cardiac surgery.

IE would embolize major arterial beds. Systemic septic emboli due to IE are common in brain, spleen, lungs, kidneys, peripheries, heart, and mesentery (45). One study involving 493 patients with IE demonstrated that septic emboli was present in 57% of patients, and about 20%–50% of cases were complicated with left-sided heart valves (46). Asymptomatic infarction (47), abscess formation (48), and splenic rupture (49) are among the splenic consequences linked to IE. About 20% of patients in the EURO-ENDO registry had splenic infarcts, which are frequently asymptomatic (50). A splenic abscess may occur in as many as 5% of cases (51). This could result from either contaminated embolic plants directly seeding in the spleen or bloodborne transmission brought on by bacteria seeding in the infected spleen location. Splenic abscesses typically do not manifest in a conventional fashion. The primary indicator is a persistent fever or bacteremia that returns during or following antibiotic therapy. It is best to have an abdominal CT, MRI, or ultrasound as soon as possible (52). For splenic infarction or antibiotic-responsive abscesses, conservative medical therapy with the right antibiotics is part of the treatment for splenic problems; however, antibiotic penetration may be low in these cases. Splenectomy is a possibility if the abscess is significant, and it is important to carefully evaluate the timing of the surgery (43). There have been prior instances of splenic infarction (25) but not splenic abscess in relation to splenic problems caused by Rothia dentocariosa IE. As far as we are aware, this is the only instance of Rothia dentocariosa IE that has been further aggravated by a splenic abscess. The patient in this report did not present with any gastrointestinal symptoms, and his splenic abscess was detected through routine abdominal ultrasonic examinations. He was in danger due to poor antibiotic treatment efficacy, underwent cardiac surgery and continued to receive anti-infection treatment. Finally, the splenic abscess was controlled without surgery.

There are currently no established treatment protocols because of the rarity of IE brought on by Rothia dentocariosa. Beta-lactam antibiotics (penicillin G, amoxicillin, or ceftriaxone), aminoglycosides, beta-lactam antibiotics plus vancomycin, and vancomycin plus gentamicin are examples of empirical therapy regimens (20, 35). The treatment lasts for a median of six weeks. The Rothia were sensitive to penicillin G or A (*n* = 36; 97%), vancomycin (*n* = 25; 84%), and rifampicin (*n* = 12; 92%), but less sensitive to gentamicin (*n* = 22; 59%) and ciprofloxacin (*n* = 10; 50%), according to the drug sensitivity data of the Ramanande and Franconieri teams. Daptomycin resistance may exist (20), and isolates resistant to penicillin and vancomycin have surfaced (53). As a result, when a treatment plan involving beta-lactam antibiotics is implemented, MIC should be determined.

According to Dustin Greve's analysis (32) of valve material samples and *in vitro* cultures of strains from IE cases of Rothia denocariosa, the bacteria were able to form fully developed bacterial biofilms. It is known that multiple mechanisms are related to the antibiotic resistance of pathogens in biofilm construction. Therefore, when treating Rothia denocariosa IE, it is advised to take into account the use of antibiotics that are efficient against biofilms. Some treatment guidelines also recommend the use of penicillin derivatives in gram-positive IE that are sensitive to penicillin and aminoglycosides in Enterococcus faecalis or Streptococcus that are resistant to penicillin. So far, it is only recommended to add biofilm-active antibiotics (i.e., rifampicin) to artificial valves associated with Staphylococcus aureus rather than natural valve IE (43, 54). Currently, it is unknown if the Rothia species is covered by these observations. In actuality, our patient began receiving active treatment as soon as Rothia dentocariosa was positively identified. However, the impact was not statistically significant when penicillin G with amikacin was applied. His blood count and CRP did not significantly drop; he continued to experience occasional fever. The therapeutic schedule was changed to ceftriaxone combined with rifampicin, which did not work well with progressive brain abscesses and spleen abscesses. The infection symptoms were partly controlled until the introduction of vancomycin and rifampicin. However, the patient finally underwent mechanical aortic valve replacement to control the infection primarily.

Depending on the patient's age, comorbidities, long-term durability, compliance with anticoagulation, and preferences, the type of valve prosthesis that should be implanted will be decided (55–57). The long-term resurgical rate after mechanical leaflet replacement is lower compared with biological leaflet replacement (56). Our patient is forty years old, free of major illnesses, and takes anticoagulants as prescribed. Therefore, replacing the mechanical valve on him is a reasonable option. Furthermore, the patient does not fit the requirements outlined in the ESC Guidelines for the use of non-mechanical leaflets in the treatment of endocarditis (43): early surgery after a recent ischaemic stroke, evidence of intracranial bleeding, woman of childbearing age, high likelihood of prolonged mechanical circulatory support, advanced age or frailty, Poor or unknown medical compliance, expected complicated and prolonged post-operative course.

## Conclusions

A rare kind of bacteria linked to IE, Rothia dentocariosa is thought to be a less dangerous agent. Nevertheless, in this case, our patient's brain and spleen abscesses-which are extremely uncommon-were caused by the uncommon bacterium infection. It is important to understand that although Rothia dentocariosa is an uncommon microbe, it can still cause infective endocarditis and lead to complicated and serious problems.

## Data Availability

The original contributions presented in the study are included in the article/Supplementary Materials, further inquiries can be directed to the corresponding author.
